# Clinical application of bronchoalveolar lavage fluid metagenomics next-generation sequencing in cancer patients with severe pneumonia

**DOI:** 10.1186/s12931-023-02654-5

**Published:** 2024-02-05

**Authors:** Chao Wang, Xiaojuan Yin, Wenqing Ma, Li Zhao, Xuhong Wu, Nan Ma, Yuepeng Cao, Quanli Zhang, Shuliang Ma, Lin Xu, Xuerong Wang

**Affiliations:** 1 Department of Critical Care Medicine, Jiangsu Cancer Hospital, Jiangsu Institute of Cancer Research, Nanjing Medical University Affiliated Cancer Hospital, Nanjing, Jiangsu 210009 China; 2 Department of Pharmacology, Nanjing Medical University, 101 Longmian Boulevard, Nanjing, Jiangsu 210029 China; 3 Department of Thoracic Surgery, Jiangsu Cancer Hospital, Jiangsu Institute of Cancer Research, Nanjing Medical University Affiliated Cancer Hospital, Nanjing, Jiangsu 210009 China; 4 Jiangsu Key Laboratory of Molecular and Translational Cancer Research, 42 Baiziting Road, Xuanwu District, Nanjing, Jiangsu 210009 China

**Keywords:** mNGS, BALF, Cancer patients, Severe pneumonia

## Abstract

**Objective:**

Metagenomic next-generation sequencing (mNGS), as an emerging technique for pathogen detection, has been widely used in clinic. However, reports on the application of mNGS in cancer patients with severe pneumonia remain limited. This study aims to evaluate the diagnostic performance of bronchoalveolar lavage fluid (BALF) mNGS in cancer patients complicated with severe pneumonia.

**Methods:**

A total of 62 cancer patients with severe pneumonia simultaneously received culture and mNGS of BALF were enrolled in this study. We systematically analyzed the diagnostic significance of BALF mNGS. Subsequently, optimization of anti-infective therapy based on the distribution of pathogens obtained from BALF mNGS was also assessed.

**Results:**

For bacteria and fungi, the positive detection rate of mNGS was significantly higher than culture method (91.94% versus 51.61%, P < 0.001), especially for poly-microbial infections (70.97% versus 12.90%, P < 0.001). Compared with the culture method, mNGS exhibited a diagnostic sensitivity of 100% and a specificity of 16.67%, with the positive predictive value (PPV) and negative predictive value (NPV) being 56.14% and 100%, respectively. The agreement rate between these two methods was 59.68%, whereas kappa consensus analysis indicated a poor concordance (kappa = 0.171). After receipt of BALF mNGS results, anti-infective treatment strategies in 39 out of 62 cases (62.90%) were optimized. Moreover, anti-tumor therapy was a high-risk factor for mixed infections (87.18% versus 65.22%, P = 0.04).

**Conclusions:**

The present study showed that cancer patients with severe pneumonia, especially those received anti-tumor therapy, were more likely to have poly-microbial infections. BALF mNGS can provide a rapid and comprehensive pathogen distribution of pulmonary infection, making it a promising technique in clinical practice, especially for optimizing therapeutic strategies for cancer patients.

**Supplementary Information:**

The online version contains supplementary material available at 10.1186/s12931-023-02654-5.

## Introduction

Pulmonary infection is a global health concern associated with high morbidity and mortality worldwide [1]. Patients with severe pulmonary infection, often arise from intertwined pathogens, require admission to the intensive care unit (ICU) and receive combination of antibiotics. Rapid and accurate identification of the pathogens is crucial for targeted antimicrobic therapy. Currently, quantitative or semi-quantitative culture of specimens obtained from BALF is the gold standard for identification of the pathogens. However, traditional culture is only used for fungal and bacterial tests, which can not meet clinical requirements due to the limitations of time consuming and low sensitivity, especially in patients with previous antibiotic exposure [2]. Hence, superior diagnostics tools for pathogen detection are necessary.

mNGS is a high-throughput nucleic acid sequencing technique that can theoretically detect all pathogens including bacteria, fungi, viruses, and parasites simultaneously in a single assay [3]. This state-of-the-art technology is characterized by unbiased, fast turn-around time, and high sensitivity [4]. Notably, the diagnostic efficiency of mNGS is less affected by antibiotics [5]. Therefore, mNGS could offer an improved identification of infectious pathogens, especially suitable for the detection of complex, rare, atypical, or slow-growing microorganisms.

Initially, mNGS was mainly used for detection of microorganisms in sterile fluids. In recent years, accumulating evidences had demonstrated the successful use of mNGS in a variety of specimens including BALF [6]. The lower respiratory tract is a relatively sterile anatomical location. BALF obtained from lower respiratory tract through bronchoscope, which is called “liquid pulmonary biopsy”, can comprehensively reflect the overall condition of the lung and be regarded as the pertinent clinical sample for pathogen evaluation in patients with respiratory infection [7]. Multiple reports have demonstrated the feasibility and effectiveness of mNGS and indicated mNGS has high diagnostic performance for BALF pathogens, especially in critically ill patients [8]. However, mNGS in pathogen detection of cancer patients with severe pneumonia has been rarely reported.

Respiratory infection occurs commonly in cancer patients, which is characterized by high frequency of multiple pathogens, unusual pathogens, and drug-resistant pathogens [9]. These pathogens present challenges for microbiological diagnosis. In this study, we thoroughly investigated the clinical information of 62 cancer patients with severe pneumonia, aimed to explore the diagnostic performance of mNGS and the pathogen distribution in the particular group of patients.

## Methods

### Patients and study design

62 cancer patients with complication-severe pneumonia, who were admitted to the ICU of the Jiangsu Cancer Hospital from September 2020 to June 2023, were enrolled. The BALF samples of all the patients were subjected to pathogen detection using both mNGS and culture methods. Inclusion criteria: (1) Patients were not limited by age or gender; (2) Cancer patients were admitted to the ICU due to severe pneumonia, which met the diagnostic criteria of the Infectious Disease Society of America/American Thoracic Society [10]; (3) Patients agreed to undergo bronchoscopy to collect BALF; (4) Clinical data was complete. Exclusion criteria: (1) Pulmonary lesions attributed to other causes; (2) Patients refused bronchoscopy and mNGS, or BALF was not simultaneously underwent routine microbiological culture; (3) Death or discharge within 48 h of ICU admission; (4) Inability to obtain all required clinical Data.

Clinical data were collected from each patient, which included age, gender, malignancy and anti-tumor therapy, comorbidities, Acute Physiology and Chronic Health Evaluation (APACHE) II score, laboratory results, treatment during the ICU stay, and patient outcomes.

### BALF collection and processing

After the patients had provided written informed consent to undergo bronchoscopy and mNGS, BALF samples were gathered by experienced clinician according to standard procedures [11]. Briefly, all patients underwent bedside bronchoscopy under intravenous combined anesthesia or topical anesthesia with 2% lidocaine. According to chest imaging, the most severe lesion sites were selected for lavage. If diffuse lesions were present, we selected the right middle lobe or the left lingual lobe as the lavage area. Subsequently, an equal volume of sterile normal saline was fractionally injected into the target bronchus and drew back after a while. The collected BALF samples were divided into two aliquotes and placed in sterile tubes. One aliquot was sent to Dinfectome Inc (Nanjing, China) for mNGS. Meanwhile, the remaining aliquot was sent to our microbiological laboratory for bacterial and fungal culture.

### mNGS of BALF

The procedure of mNGS for BALF samples includes nucleic acid extraction, library construction, sequencing, and information analysis.

### Nucleic acid extraction

DNA was extracted from BALF using the Tiangen Magnetic DNA Kit (Tiangen) according to the manufacturer’s instructions, and human host DNA was removed using Benzonase (Qiagen) and Tween20 (Sigma). The total extracted DNA was quality assessed by Qubit (Thermo Fisher Scientific) and NanoDrop (Thermo Fisher Scientific).

### Library construction and sequencing

The extracted DNA was ultrasonicated into 50–100 bp fragments. DNA libraries were then constructed through terminal repair, adapter ligation, and PCR amplification, using the KAPA Hyper Prep Kit (KAPA Biosystems). Agilent 2100 Bioanalyzer and Qubit were used for quality control of the DNA libraries. Subsequently, qualified DNA libraries were sequenced (50 bp, single-end) on the DIFSEQ-200 platform.

### Bioinformatic analysis

Initially, raw data were preprocessed by Trimmomatic to remove low-quality reads, contaminative adapter, duplicate reads, as well as short reads (length < 35 bp) and obtain high-quality sequencing data. Subsequently, human host sequences were recognized and excluded by mapping against the human reference genome (hs37d5) using Bowtie2 software. Finally, the remaining sequences were aligned to current bacterial, viral, fungal, and parasitic microbial genome databases using Kraken2 software. Meanwhile, the relative abundance of each species was calculated using the Bracken software. The Microbial Genome Databases were downloaded from GenBank (http://ftp.ncbi.nlm.nih.gov/genomes/genbank/).

### Statistical analysis

All statistical analyses were performed by SPSS 27.0 software (IBM Corp., Armonk, NY, USA). Continuous variables were expressed as the median with interquartile ranges due to the non-normal distribution and compared by rank-sum test. Categorical variables were displayed as number and percentage and compared by the chi-square test. A 2 × 2 contingency table was applied to determine the sensitivity, specificity, PPV and NPV of mNGS. The diagnostic performance was compared by the McNemar test and the test concordance was assessed using the kappa statistic. All tests were two-tailed, and p < 0.05 denoted statistically significant.

## Results

### Patient characteristics

Between September 2020 to June 2023, 62 cancer patients with severe pneumonia were enrolled in this study, including 56 males (90.32%) and 6 females (9.68%), with a median age of 60 years, and the majority were above 60 years old (83.87%). All patients were diagnosed with malignant tumors, including 22 cases of esophagus carcinoma, 21 cases of lung carcinoma, 5 cases of lymphoma, 3 cases of colorectal carcinoma, 2 cases of nasopharynx carcinoma, 1 case each of gastric carcinoma, breast carcinoma, bladder carcinoma, tonsillar carcinoma, thymic carcinoma, oral malignancy, mediastinal malignancy, malignant pleural mesothelioma, and 1 case of metastatic lymph node malignancy which the primary tumor was not identified. Among them, a total of 13 patients (20.97%) had myelosuppression (Table S1) due to anti-tumor therapy.

The median APACHE II score was 18, and the median oxygenation index was 120.8 mmHg. In addition, 48 patients (77.42%) received invasive mechanical ventilation, and 17 patients (27.42%) developed septic shock. Despite aggressive treatment, the total 30-day mortality reached 43.55%, suggesting that cancer patients with severe pneumonia were characterized by rapid progression, serious illness, difficult treatment and high mortality. The detailed clinical information of recruited patients including demographic characteristic, laboratory findings and prognosis was presented in Table 1.

**Table 1:** Clinical characteristics of 62 enrolled patients

Characteristic	Value (median (IQRs) or no. (%))
**Gender**	
Male	56 (90.32%)
Female	6 (9.68%)
**Age (years)**	69.5 (63.5, 74)
< 60	10 (16.67%)
≥ 60	52 (83.87%)
**Malignancy**	
Esophagus cancer	22 (35.48%)
Lung cancer	21 (33.87%)
Lymphoma	5 (8.06%)
Colorectal cancer	3 (4.84%)
Nasopharynx cancer	2 (3.23%)
other malignancies	9 (14.52%)
**Comorbidity**	
Diabetes mellitus	13 (20.97%)
Myelosuppression	13 (20.97%)
Digestive tract fistula	9 (14.52%)
**Disease severity**	
APACHE II score	18 (14.25, 24)
Oxygenation index (mmHg)	120.8 (88.675, 158.125)
Invasive mechanical ventilation	48 (77.42%)
Septic shock	17 (27.42%)
**Inflammation biomarker**	
White blood cells (10^9^/L)	10.97 (7.0625, 15.975)
Percentage of neutrophil (%)	89.3 (82.9, 92.625)
C reactive protein (mg/L)	137.505 (72.825, 185.4225)
Procalcitonin (ng/mL)	1.85 (0.389, 7.4265)
**Outcomes**	
Total 30-day mortality	27 (43.55%)

*Abbreviations*: APACHE II, acute physiology and chronic health evaluation II; IQR, interquartile range

### Comparison and concordance of mNGS and culture method

Since culture is only used for determine of a limited scale of fungi and bacteria, we compared the BALF mNGS results in severe pneumonia patients with a paired BALF culture results for bacteria and fungi. In this study, the positive rate of mNGS was significantly higher than culture method (91.94% versus 51.61%, P < 0.001). Compared with the culture method, mNGS exhibited a diagnostic sensitivity of 100% and a specificity of 16.67%, with the PPV and NPV being 56.14% and 100%, respectively (Table 2).

**Table 2:** Diagnostic performance and concordance of mNGS relative to culture method in cancer patients with severe pneumonia

	Culture +		Culture -	Total	Diagnostic performance of mNGS			
mNGS +	32		25	57	Sensitivity% (95% CI)	Specificity% (95% CI)	PPV% (95% CI)	NPV% (95% CI)
mNGS -	0		5	5	100 (86.66–100)	16.67 (6.30–35.45)	56.14 (42.43–69.02)	100 (46.29–100)
Total	32		30	62				
**Consensus analysis**								
P value		0.016						
Kappa		0.171						

*Abbreviations*: mNGS, metagenomic next-generation sequencing; CI, confidence interval; PPV, positive predictive value; NPV, negative predictive value

**Fig. 1:**
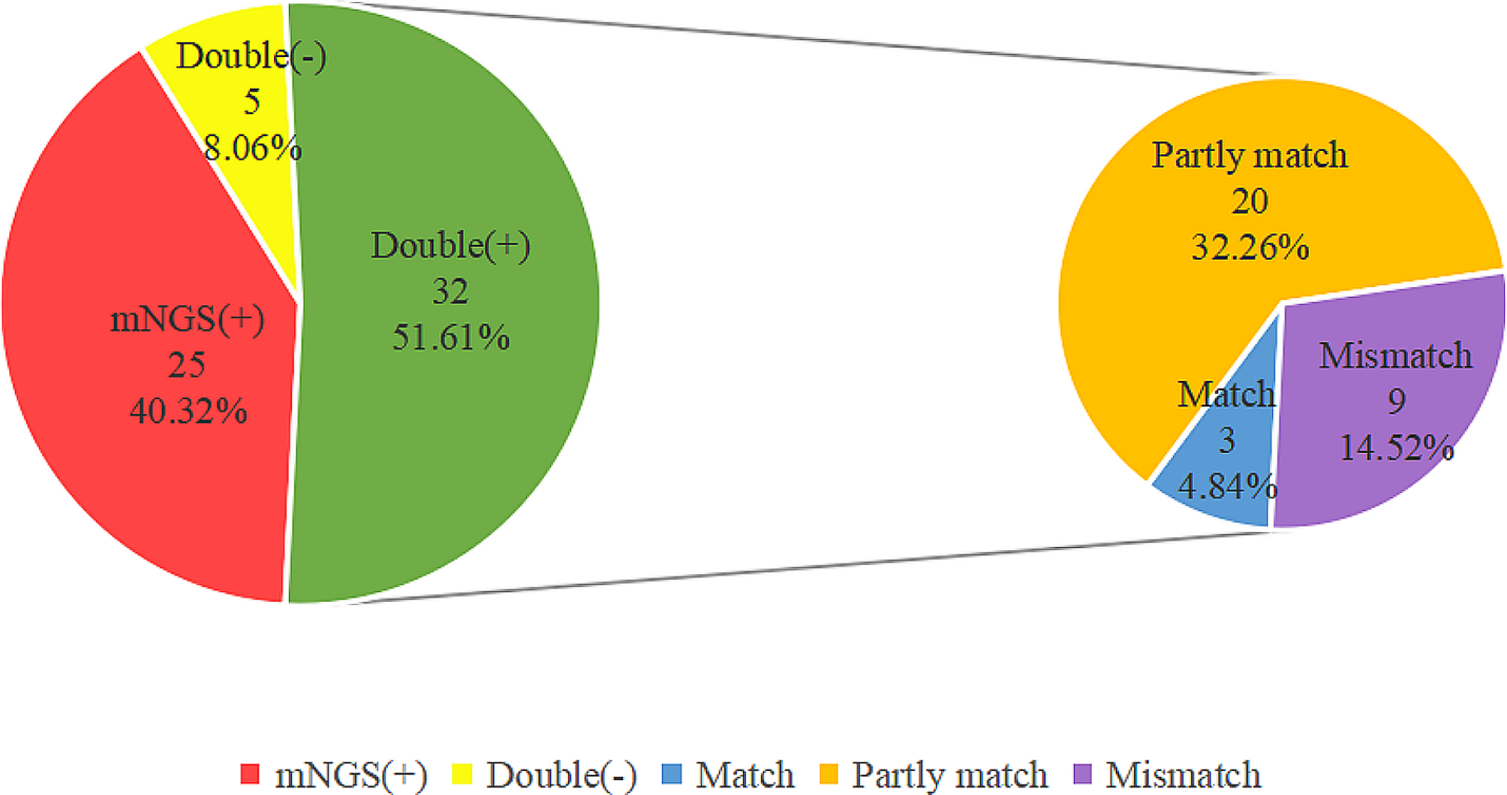
Concordance between mNGS and culture method. The results of mNGS and culture method were double positive in 32 (51.61%) cases, double negative in 5 (8.06%) cases, and only mNGS positive in 25 (40.32%) cases. For the double positive subset, 3 (4.84%) cases were consistent, 20 (32.26%) cases were partially consistent, and 9 (14.52%) cases were completely inconsistent. *Abbreviations*: mNGS: metagenomic next-generation sequencing

Moreover, among the 62 severe pneumonia patients, mNGS and culture method were both positive in 32 (51.61%) cases and both negative in 5 (8.06%) cases, with an agreement rate of 59.68% between these two methods. Further consensus analysis showed a kappa value of 0.171, indicating a poor concordance between the two methods (Table 2). The remaining 25 (40.32%) cases were positive by mNGS only and positive cases by culture method only were not observed in this study. Within the 32 double-positive cases, the results of mNGS and culture method were completely matched in 3 (4.84%) cases, partially matched in 20 (32.26%) cases and mismatched in 9 (14.52%) cases (Fig. 1).

Overall, mNGS identified more bacteria (163 versus 30) and fungi (47 versus 13) than culture method (Figure S1). Furthermore, poly-microbial infections (two or more microorganisms) were identified significantly more frequently in mNGS than culture method (70.97% versus 12.90%, P < 0.001). Among 30 patients with negative culture results, mNGS produced mono-microbial detection in 8 cases and poly-microbial detection in 17 cases. A total of 65 strains of bacteria and 18 fungi were identified by mNGS, indicated mNGS raised the efficiency of pulmonary infection diagnosis. With regard to the double positive patients, all 3 completely matched cases were mono-microbial infections. When examining the partially matched and mismatched cases, more bacteria (96 versus 28), more fungi (28 versus 12), and more poly-microbial infections (27 versus 8) were identified by mNGS than culture method (Figure S2), suggested even in the patients with positive culture results, mNGS still played an important role of identifying more and rare pathogens. In conclusion, these results highlighted the complex etiologies of severe pneumonia in cancer patients and the ability of mNGS to detect all of them efficiently.

### Pathogens detected by mNGS and clinical impact of mNGS results on treatment

**Fig. 2:**
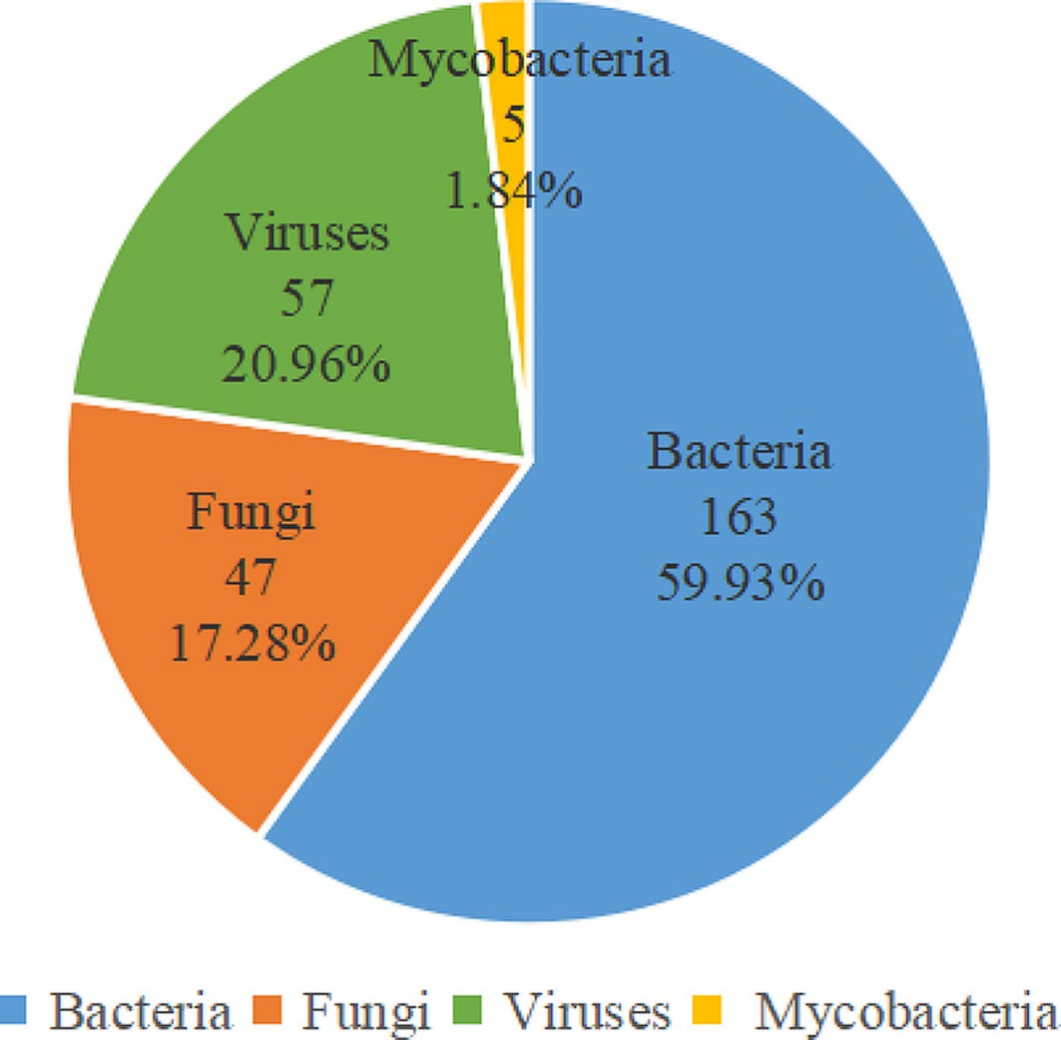
Proportion of various pathogens identified by mNGS. Bacteria were the most frequently pathogens detected by mNGS (59.93%), followed by viruses (20.96%), fungi (17.28%), and mycobacteria (1.84%)

**Fig. 3:**
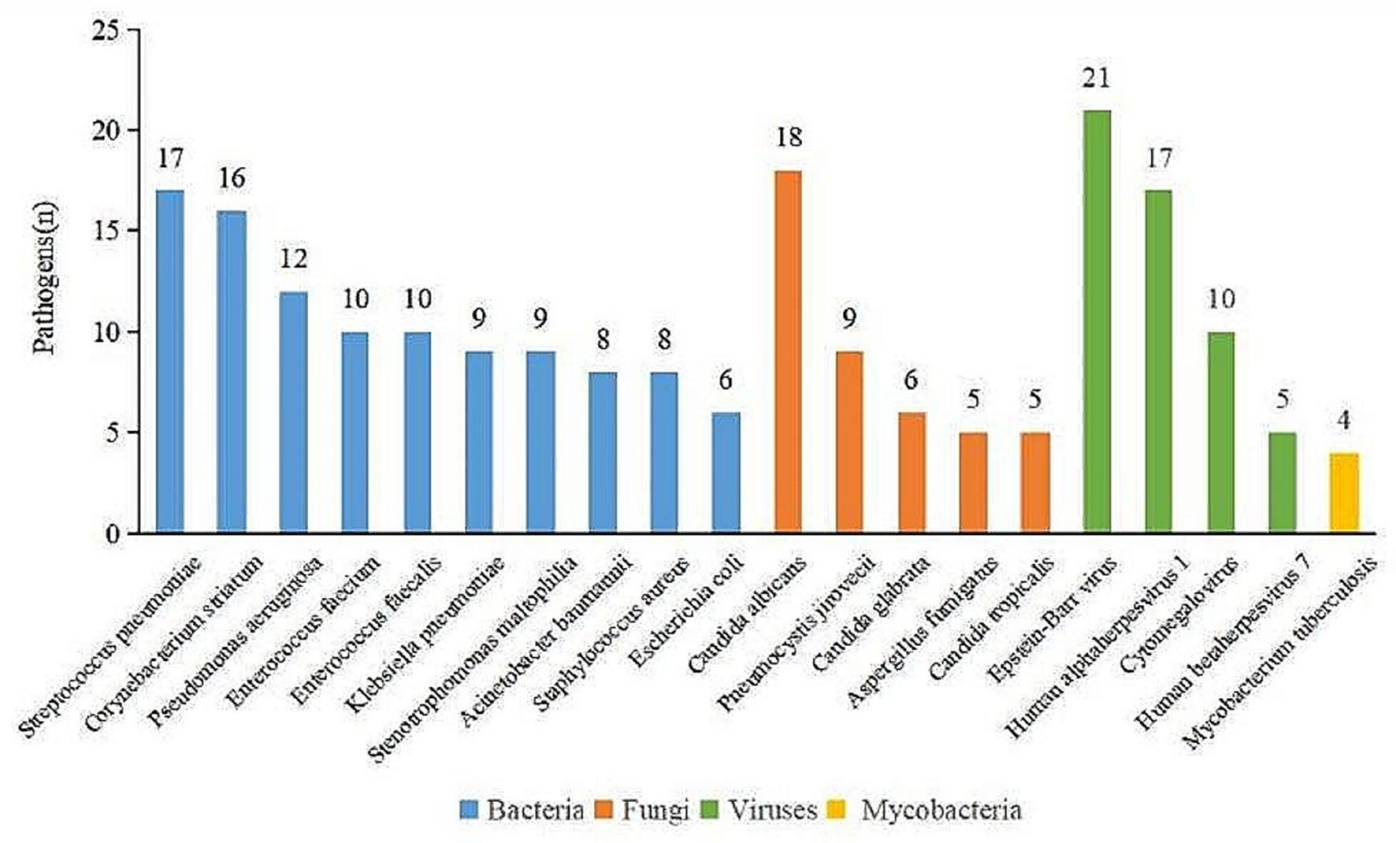
Number of pathogens identified by mNGS. The most common bacteria, fungi, viruses, and mycobacteria were *Streptococcus pneumoniae*, *Candida albicans*, *Epstein-Barr virus*, and *Mycobacterium tuberculosis* respectively

Then we analyzed strains of pathogen information provided by mNGS in order to identify the most popular pathogenic microorganism, and then to provide valuable comments for optimizing therapy. A total of 272 strains of pathogens were identified in 59 cases by mNGS. The most frequently detected pathogens were bacteria (59.93%), followed by viruses (20.96%), fungi (17.28%), and mycobacteria (1.84%) (Fig. 2). As the most frequently detected pathogens, 163 bacteria were identified in 50 patients, of which the top ten most common bacteria were *Streptococcus pneumoniae* (17 cases), *Corynebacterium striatum* (16 cases), *Pseudomonas aeruginosa* (12 cases), *Enterococcus faecium* (10 cases), *Enterococcus faecalis* (10 cases), *Klebsiella pneumoniae* (9 cases), *Stenotrophomonas maltophilia* (9 cases), *Acinetobacter baumannii* (8 cases), *Staphylococcus aureus* (8 cases), and *Escherichia coli* (6 cases) (Fig. 3). Correspondingly, 30 bacteria were identified in 23 patients by culture method, with the top three being *Pseudomonas aeruginosa* (6 cases), *Acinetobacter baumannii* (5cases), and *Stenotrophomonas maltophilia* (4 cases) (Figure S3). A total of 47 fungi were identified in 34 patients, and the most common fungus was *Candida albicans* (18 cases), followed by *Pneumocystis jirovecii* (9 cases), *Candida glabrata* (6 cases), *Aspergillus fumigatus* (5 cases), and *Candida tropicalis* (5 cases) (Fig. 3). However, culture method only detected 13 fungi, and the vast majority of them were *Candida albicans* (12 cases). Meanwhile, 57 viruses were confirmed in 32 patients. *Epstein-Barr virus* (21 cases), *Human alphaherpesvirus 1* (17 cases), and *Cytomegalovirus* (10 cases) ranked the first three in turn (Fig. 3). BALF mNGS also identified 5 mycobacteria, among them *Mycobacterium tuberculosis* were the main strains (Fig. 3).

**Fig. 4:**
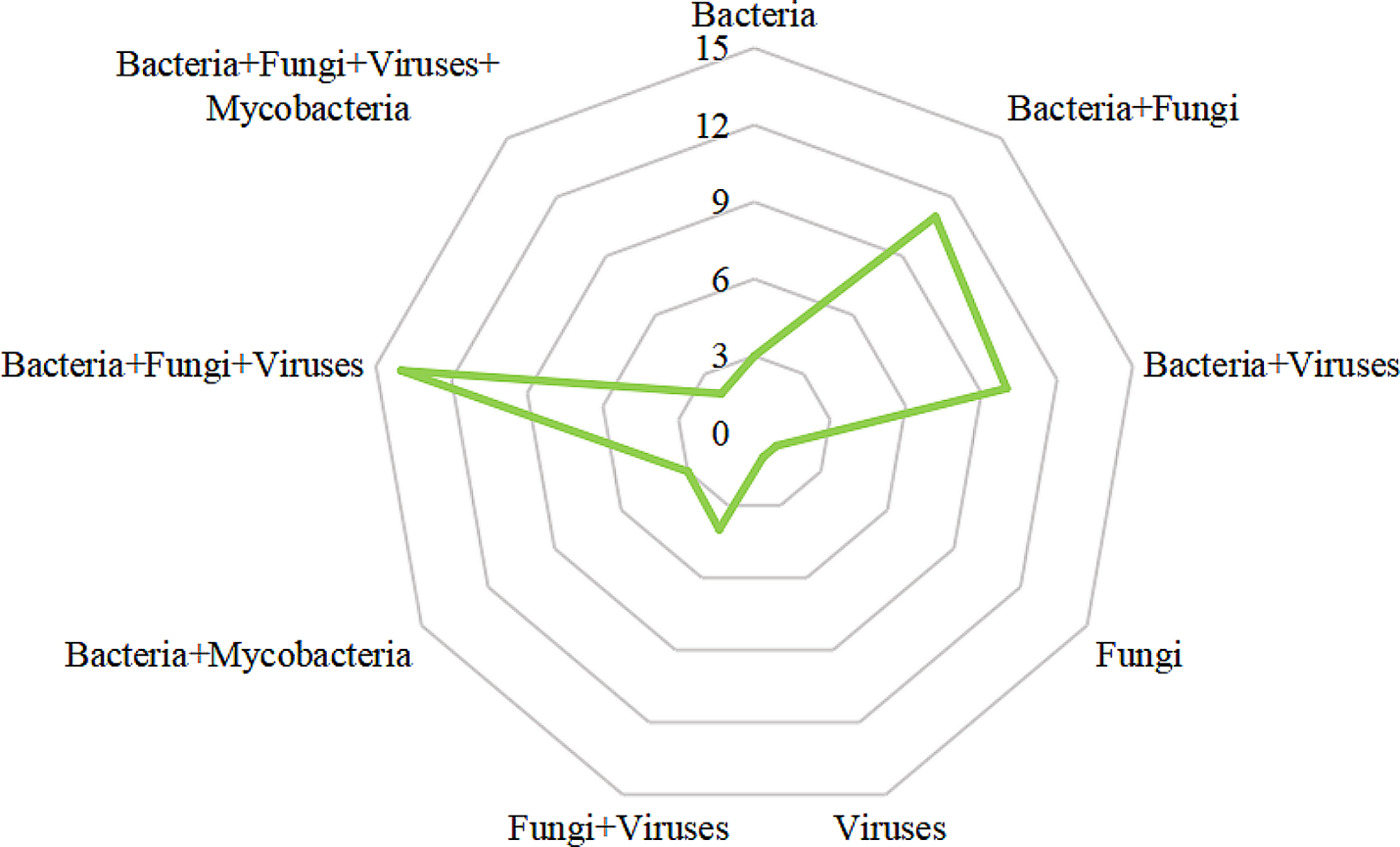
Distribution of patients with poly-microbial infection. Bacteria-fungi-viruses, bacteria-fungi, and bacteria-viruses were the top three co-pathogens

According to the mNGS results, a total of 49 (79.03%) were diagnosed as poly-microbial infection. Among the poly-microbial infections, bacteria-fungi-viruses (14 cases), bacteria-fungi (11 cases), and bacteria-viruses (10 cases) were the top three co-pathogens (Fig. 4).

In this study, all patients received empiric antibiotic therapy and antibiotics were adjusted after receiving the microbiological test results. In total, 39 cases (62.90%) received adjusted treatment owing to BALF mNGS results, and the remaining 23 cases (37.10%) continued the previous treatment. Because prior empirical antibiotics have covered the detected pathogens (Table S2). Notably, among the cases receiving change in treatment, mNGS identified multiple types of pathogens requiring different targeted treatments. As a consequence, adding or adjusting antibiotics were always needed, especially combining anti-fungal treatment. In addition, the turnaround time of mNGS was relatively stable, usually about 24 to 48 h. By rapidly optimizing anti-infective treatment, pulmonary infection improved in more than half of patients within the subsequent 7 days. The evidence showed that white blood cell count, C reactive protein, and procalcitonin decreased in 56.45%, 66.13%, and 62.90% of patients, respectively, oxygenation index increased in 80.65% of patients, and chest X-ray or CT improved in 51.61% of patients (Table S3). In summary, cancer patients with severe pneumonia were prone to poly-microbial infection. mNGS provided more comprehensive and timely etiological results, leading to prompt changes in the anti-infective therapy at very early stage and benefit patients.

### Effect of anti-tumor therapy on the distribution of pathogens

To explore the effect of anti-tumor therapy on the distribution of microbiome, we divided the patients into two groups, patients have received anti-tumor therapy, including chemotherapy, targeted therapy, immunotherapy and/or radiotherapy, were included in the observation group (39 cases). Correspondingly, the control group represented patients have not received anti-tumor therapy or only underwent surgery (23 cases).

There were no significant differences in median age and disease severity (APACHE II score, oxygenation index, invasive mechanical ventilation, and septic shock) between the two groups. However, the total 30-day mortality was significantly higher in the anti-tumor therapy group than the control group (p = 0.033). In terms of inflammation biomarker, C reactive protein, procalcitonin, and percentage of neutrophil did not differ significantly between the two groups, while white blood cell count in the anti-tumor therapy group was significantly lower than the control group (p = 0.001), which was correlated with the existence of myelosuppression in these patients (Table 3).

**Fig. 5:**
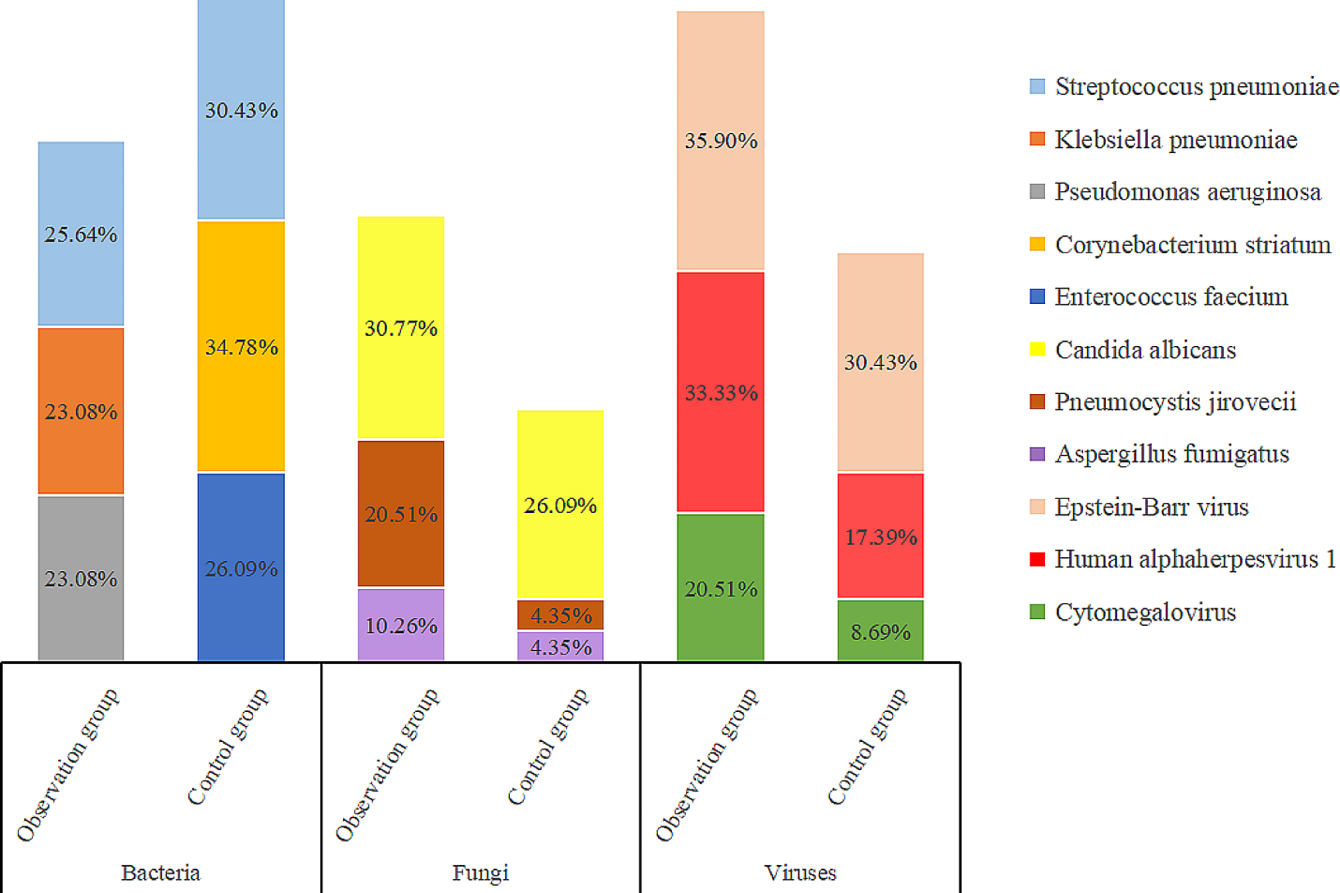
The most common pathogens detected by mNGS in observation group and control group. In the observation group, *Streptococcus pneumoniae*, *Klebsiella pneumoniae*, and *Pseudomonas aeruginosa* ranked the were the top three bacteria, whereas in the control group, *Corynebacterium striatum*, *Streptococcus pneumoniae*, and *Enterococcus faecalis* were the top three bacteria. In both groups, the most common fungi were *Candida albicans, Pneumocystis jirovecii* and *Aspergillus fumigatus*, and the most common viruses were *Epstein-Barr virus*, *Human alphaherpesvirus 1*, and *Cytomegalovirus*. *Definition*: The observation group represented patients have received anti-tumor therapy. The control group represented patients have not received anti-tumor therapy or only underwent surgery

**Fig. 6:**
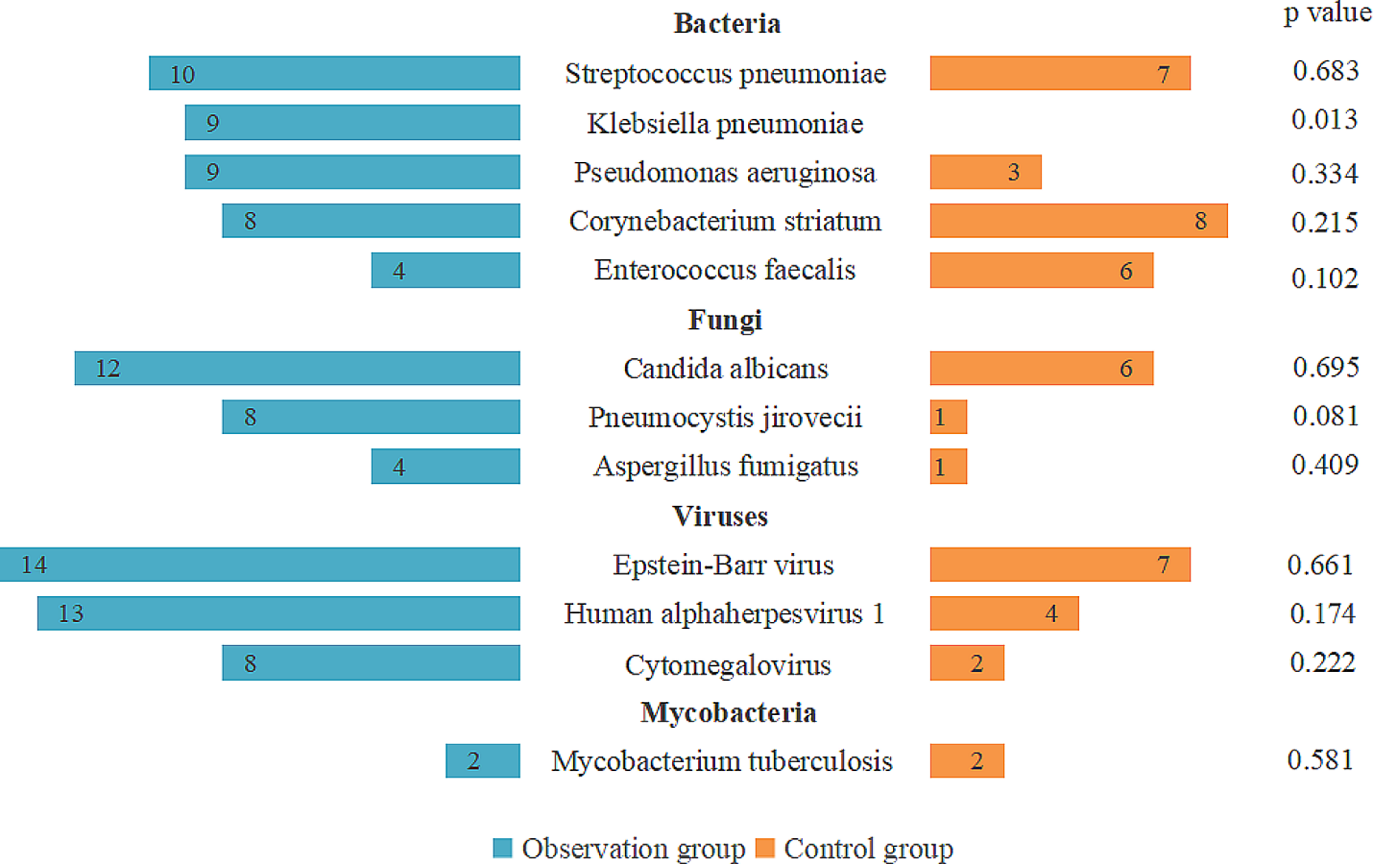
Species distribution of pathogens detected by mNGS in observation group and control group. *Klebsiella pneumoniae* was detected only in the observation group, while the proportion of other pathogenic species did not differ between the two groups. *Definition*: The observation group represented patients have received anti-tumor therapy. The control group represented patients have not received anti-tumor therapy or only underwent surgery

Furthermore, mNGS respectively identified 190 and 82 strains of pathogens in both groups. No significant differences in the proportion of each pathogenic species between the two groups were observed, nor in the detection rate of drug resistance gene. Nevertheless, poly-microbial infections were identified significantly more frequently in the anti-tumor therapy group than the control group (p = 0.04) (Table 3). In the anti-tumor therapy group, *Streptococcus pneumoniae*, *Klebsiella pneumoniae*, and *Pseudomonas aeruginosa* ranked the first three in turn, whereas in the control group, *Corynebacterium striatum*, *Streptococcus pneumoniae*, and *Enterococcus faecalis* were the top three bacteria. The most common fungus was *Candida albicans* in both groups, followed by *Pneumocystis jirovecii* and *Aspergillus fumigatus*. The top three viruses in both groups were also the same, which were *Epstein-Barr virus*, *Human alphaherpesvirus 1*, and *Cytomegalovirus*, respectively (Fig. 5). Although mNGS reported different detection rates of pathogens in the two groups, for instance, 8 cases of *Pneumocystis jirovecii* (20.51%) and 4 cases of *Aspergillus fumigatus* (10.26%) were detected in the anti-tumor therapy group, while there was only one for each (4.35%) in the control group. However, except for *Klebsiella pneumoniae*, the distribution of each microbiological species did not differ significantly between the two groups (Fig. 6). Overall, anti-tumor therapy may affect the distribution of pathogens in severe pneumonia, leading to more poly-microbial infections and higher mortality.

**Table 3:** Clinical characteristics of the two groups patients. The observation group represented patients have received anti-tumor therapy. Correspondingly, the control group represented patients have not received anti-tumor therapy or only underwent surgery

Characteristic(median or no. (%))	Observation group (n = 39)	Control group (n = 23)	p value
Age (years)	70 (62, 76)	69 (63.5, 73.5)	0.901
APACHE II score	19 (15.5, 25.5)	16 (14, 20.5)	0.095
Oxygenation index(mmHg)	128 (87.35, 165.05)	119.5 (98.5, 133.8)	0.565
Invasive mechanical ventilation	29 (74.36%)	19 (82.60%)	0.453
Septic shock	12 (30.77%)	5 (21.74%)	0.441
Total 30-day mortality	21 (53.85%)	6 (26.09%)	0.033
**Inflammation biomarker**			
White blood cells (10^9^/L)	9.27 (6.555, 13.14)	15.58 (10.93, 20.06)	0.001
Percentage of neutrophil (%)	88 (82, 92.9)	90.1 (88.1, 92.3)	0.386
C reactive protein (mg/L)	134.77 (73.26, 188.46)	139.61 (77.665, 185.165)	0.867
Procalcitonin (ng/mL)	1.83 (0.396, 5.205)	1.2 (0.2465, 4.805)	0.304
**BALF-mNGS results**			
Bacteria	108 (56.84%)	55 (67.07%)	0.114
Fungi	36 (18.95%)	11 (13.41%)	0.268
Viruses	43 (22.63%)	14 (17.07%)	0.301
Mycobacteria	3 (1.58%)	2 (2.44%)	0.628
Poly-microbial infection	34 (87.18%)	15 (65.22%)	0.040
Drug resistance gene	13 (33.33%)	4 (17.39%)	0.174

*Abbreviations*: APACHE II, acute physiology and chronic health evaluation I

## Discussion

Cancer patients are at a particular risk for pulmonary infection by a wide variety of different pathogens, often progressing to severe pneumonia rapidly. Due to the refractory and high lethality, quick and accurate pathogen diagnosis has become the key to treatment but is also a clinical challenge. Fortunately, a new state-of-the-art technology, mNGS has been widely used in pathogen detection in recent years. Although many researches have evaluated the performance of BALF mNGS in pulmonary infection of different patient populations, cancer patient population represented usually less than 30% of the patients enrolled [12–15]. This study is specifically designed to evaluate the diagnostic performance of BALF mNGS in cancer patients with severe pneumonia.

BALF was taken for mNGS and culture due to its simple acquisition through bronchoscopy and high reliability for pathogen detection in pulmonary infection. In this study, we explored the diagnostic performance of BALF mNGS in cancer patients with severe pneumonia. Our study showed that overall positive rate of mNGS was significantly higher than culture method. In addition, mNGS identified more bacteria, more fungi, and more poly-microbial infections. These features are in line with previous research [16], suggesting the strong diagnostic utility of mNGS, especially in describing poly-microbial ecosystems.

According to our study, mNGS had a diagnostic sensitivity of 100% in identification of bacteria and fungi compared with culture method. As previous research showed that the diagnostic sensitivity of mNGS in severe pulmonary or immunocompromised patients was significantly higher than ordinary patients [8]. All patients enrolled were confirmed severe pneumonia with cancer, which partly accounted for the result in our study. On the contrary, using the culture-based results as the reference standard to evaluate the diagnostic performance of mNGS, the specificity of mNGS might be underestimated attribute to the relatively high false-negative rate of traditional etiological culture. Meanwhile, this study also elaborated the consistency between mNGS and culture method in 37 cases with a consistent rate of 59.68% (32 double positive and 5 double negative). Different from previous studies [17], consensus analysis showed a poor concordance between the two methods. Potential explanations for above results include the following: Firstly, the efficacy of culture assay was hampered by prior antibiotic exposure before sampling [18], but mNGS was less affected. Secondly, the presence of rare and fastidious pathogens in cancer patients also reduced the sensitivity of culturing method. Thirdly, the competition between certain microorganisms made pathogen culture even more difficult [19]. Hence, it may require many other clinical auxiliary inspections such as Gram staining, serum immunological test, G test, GM test, and PCR tests, to further evaluate the diagnostic performance of mNGS.

In the present study, BALF mNGS comprehensively revealed pathogen distribution in severe pneumonia of cancer patients. According to the data, mNGS showed remarkable advantage in detecting pathogens that had low yield by regular culture, including *Streptococcus pneumoniae*, *Pneumocystis jirovecii*, and *Aspergillus*. Besides, most cases were poly-microbial infections detected by mNGS probably attributed to the immunosuppression in cancer patients. Thus, combined therapies were usually required due to different antibiotic spectrums.

After obtaining the BALF mNGS pathogenic microorganism reports, more than half patients adjusted treatment strategies and received more targeted antimicrobial treatment, especially against fungal infection. *Pneumocystis jirovecii* was an opportunistic fungal in immunocompromised patients [20] and occurred in almost half of this special population with pulmonary infection based on a previous research [21]. In our study, mNGS reported *Pneumocystis jirovecii* as a common fungus, indicated that Pneumocystis pneumonia has become an emerging trend in cancer patients. Similar to the previous study [22], our results highlighted the clinical utility of mNGS in the detection of *Pneumocystis jirovecii*, thus solved the problem of unable to be cultured. *Aspergillus* was also a common pathogen in cancer patients with pulmonary infection, which culturing was time-consuming with low positive rate. Past studies suggested that it was a challenge for mNGS to identify *Aspergillus* due to the low efficiency of DNA extraction from the thick and tough fungal cell wall [23]. Recently, with the optimization of DNA extraction method, the diagnostic performance of mNGS for *Aspergillus* was improved [24, 25]. Similarly, our findings demonstrated that mNGS had outstanding performance in the diagnosis of *Aspergillus*, with a total of 7 cases of *Aspergillus* identified by mNGS, including 5 cases of *Aspergillus fumigatus*, 1 case of *Aspergillus nidulans*, and 1 case of *Aspergillus flavus*, while only 1 case of *Aspergillus fumigatus* by culture testing. Besides, mNGS also identified 3 cases of *Legionella pneumophila*. Because of the difficulty of detection by culture method and the resistance to empiric β-lactam therapy, *Legionella* is often classified as an atypical pathogen [26]. Therefore, a rapid diagnosis by mNGS is essential for the treatment of Legionella pneumonia. In clinical practice, mNGS provides clinicians with reliable microbiological information concerning pulmonary infection and guides clinicians regarding antimicrobial treatment, especially for those uncultured pathogens, such as trimethoprim-sulfamethoxazole for *Pneumocystis jirovecii*, voriconazole for *Aspergillus*, and roxithromycin for *Legionella*.

On the other hand, the treatment strategies were not adjusted for 23 cases. Mostly because broad-spectrum antibiotics have been used empirically and mNGS did not detect additional pathogens. In addition, considering the serious condition, we continued the empiric treatment for patients with negative results of mNGS. Despite the high sensitivity, negative results of mNGS can help to rule out pulmonary infection, but missed diagnoses may also exist [27]. In the face of negative results of mNGS, antibiotic de-escalation or discontinuation should be cautious, unless routine microbiological tests were also negative and patients were clinically improving without signs of active infection.

After adjustment of anti-infective treatment, inflammation biomarker, oxygenation index, and chest X-ray or CT improved in more than half of patients within the subsequent 7 days, whereas 30-day mortality was still not optimistic. These findings indicated mNGS may be beneficial for pulmonary infection control at the early stage and provide a greater therapeutic opportunity to cancer patients with severe pneumonia. However, the influence of mNGS on prognosis of these patients, especially the long-term prognosis, needs further evaluation.

In addition to the heterogeneity of cancer patients, anti-tumor therapy, with its immunosuppressive effects such as profound neutropenia, leads to the complicacy of pathogenic microorganism in patients with severe pneumonia. Our study showed that patients received anti-tumor therapy were more likely to have mixed infections, highly suggested the urgently needing of mNGS in these special group of patients to help guide the subsequent anti-infective treatment. Patients received anti-tumor therapy have a tendency of high fungal detection rate in spite of no significant differences in this study. Of particular note are *Pneumocystis jirovecii* and *Aspergillus*, as anti-tumor therapy is a high-risk factor for pneumocystis jirovecii pneumonia (PJP) [28] and invasive pulmonary aspergillosis (IPA) [29].

In spite of these unique diagnostic advantages, some disadvantages are inherent to mNGS. First, there is currently no unified standard guiding the implementation of mNGS. Second, as a highly sensitive test method, mNGS is incapable of distinguishing contamination or colonization from disease-causing pathogens [30]. Third, mNGS is limited to DNA sequencing, resulting in a lack of RNA virus detection. Fourth, although mNGS can provide some susceptibility information, drug sensitivity test is still dependent on culture method. Fifth, the relatively high cost, particularly not included in national health insurance, considerably limits the clinical application of mNGS. As an imperfect approach, mNGS is a supplementary, but not a substitute for conventional culture method.

This study also contained certain limitations. Firstly, as a single-center study with a small sample size, potential selection bias was inevitable. Additionally, due to the constraints of laboratory conditions, many other clinical auxiliary inspections were not included in our study, resulting in loss of useful pathogenic microorganism information, especially for virus infection. Furthermore, previous anti-infective therapy could alter pathogen distribution. However, we did not exclude patients with a history of antimicrobial therapy, which might skew the results. Finally, we did not evaluate the influence of mNGS on prognosis of patients. Prospective, multi-center, and large-scale studies are required to evaluate the diagnostic performance and clinical application of mNGS.

## Conclusion

In summary, our findings support that there are complicated pathogen co-infections in cancer patients with severe pneumonia. BALF mNGS is able to rapidly and globally identify pathogens and provide strong evidence for efficient anti-infective treatment, making it a promising technique for microbiological detection. Therefore, we strongly recommend early application of BALF mNGS in cancer patients with severe pneumonia. In addition, combined use of mNGS and traditional culture methods could increase the specificity of diagnosis for infectious disease.

### Electronic supplementary material

Below is the link to the electronic supplementary material.

**Supplementary Material 1:** **Table S1.** Diagnostic and grading criteria for myelosuppression. According to WHO classification standard of common toxic and side effects of anticancer drugs. **Table S2.** Clinical impact of mNGS results on anti-infective treatment. **Table S3.** Changes in patient indicators within the subsequent 7 days after optimizing anti-infective treatment

**Supplementary Material 2:** **Figure S1.** The comparison of detected results between mNGS and culture method. mNGS identified more bacteria (163 versus 30) and fungi (47 versus 13) than culture method. **Figure S2.** Comparison of pathogens detected by mNGS and culture method in the double positive patients. **Figure S3.** Number of pathogens identified by culture method. The the top three were *Pseudomonas aeruginosa*, *Acinetobacter baumannii*, and *Stenotrophomonas maltophilia*. The most common fungus was *Candida albicans*

## Data Availability

The data and materials in the current study are available from the corresponding author upon reasonable request.

## Abbreviations

mNGS: Metagenomic next-generation sequencing
BALF: bronchoalveolar lavage fluid
PPV: positive predictive value
NPV: negative predictive value
ICU: intensive care unit
APACHE II score: Acute Physiology and Chronic Health Evaluation II score
PJP: pneumocystis jirovecii pneumonia
IPA: invasive pulmonary aspergillosis
