# Supplementary material for: Clinical application of bronchoalveolar lavage fluid metagenomics next-generation sequencing in cancer patients with severe pneumonia
Source: Respir Res. 2024 Feb 5;25:68. doi: 10.1186/s12931-023-02654-5 (PMC10840150; doi:10.1186/s12931-023-02654-5)

Figure S1 The comparison of detected results between mNGS and culture method. mNGS identified more bacteria (163 versus 30) and fungi (47 versus 13) than culture method.


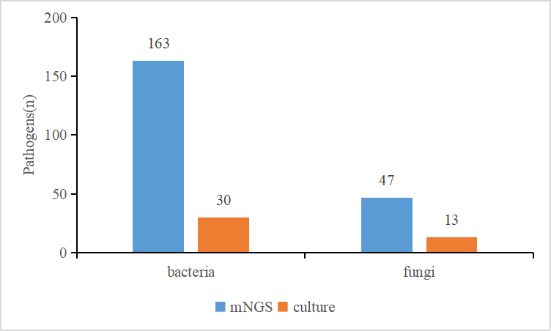


Figure S2 Comparison of pathogens detected by mNGS and culture method in the double positive patients.


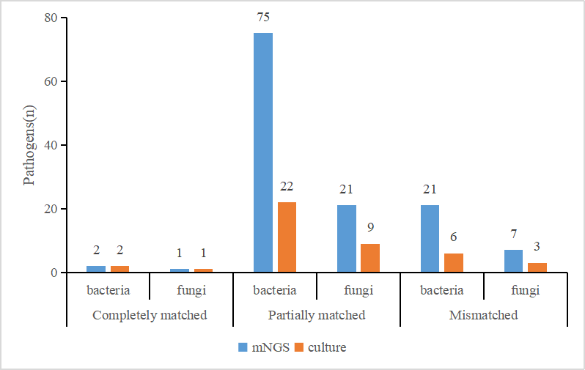


Figure S3 Number of pathogens identified by culture method. The the top three were *Pseudomonas aeruginosa*, *Acinetobacter baumannii*, and *Stenotrophomonas maltophilia*. The most common fungus was *Candida albicans.*


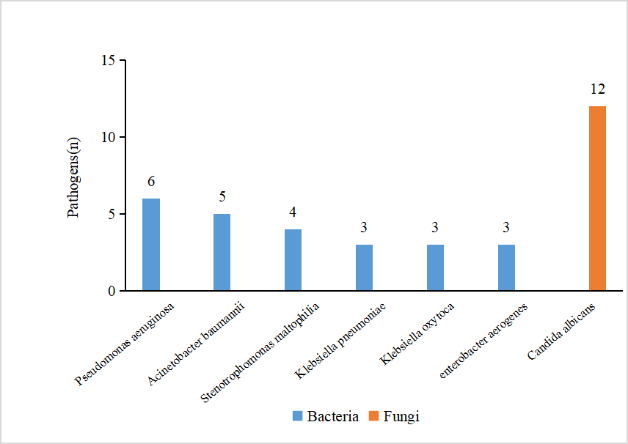

Supplement: Supplementary file 1 — Supplementary Material 1: Table S1. Diagnostic and grading criteria for myelosuppression. According to WHO classification standard of common toxic and side effects of anticancer drugs. Table S2. Clinical impact of mNGS results on anti-infective treatment. Table S3. Changes in patient indicators within the subsequent 7 days after optimizing anti-infective treatment [file 12931_2023_2654_MOESM1_ESM.docx]
