# Supplementary material for: Clinical application of bronchoalveolar lavage fluid metagenomics next-generation sequencing in cancer patients with severe pneumonia
Source: Respir Res. 2024 Feb 5;25:68. doi: 10.1186/s12931-023-02654-5 (PMC10840150; doi:10.1186/s12931-023-02654-5)
Supplement: Supplementary file 2 — Supplementary Material 2: Figure S1. The comparison of detected results between mNGS and culture method. mNGS identified more bacteria (163 versus 30) and fungi (47 versus 13) than culture method. Figure S2. Comparison of pathogens detected by mNGS and culture method in the double positive patients. Figure S3. Number of pathogens identified by culture method. The the top three were Pseudomonas aeruginosa, Acinetobacter baumannii, and Stenotrophomonas maltophilia. The most common fungus was Candida albicans [file 12931_2023_2654_MOESM2_ESM.docx]

Table S1 Diagnostic and grading criteria for myelosuppression. According to WHO classification standard of common toxic and side effects of anticancer drugs.

| Classification index | Leukocyte  (10^9/L) | Granulocyte  (10^9/L) | Thrombocyte  (10^9/L) | Hemoglobin  (g/L) |
| --- | --- | --- | --- | --- |
| Level zero | ≥4 | ≥2 | ≥100 | ≥110 |
| Level one | 3.9-3.0 | 1.9-1.5 | 99-75 | 109-95 |
| Level two | 2.9-2.0 | 1.4-1.0 | 74-50 | 94-80 |
| Level three | 1.9-1.0 | 0.9-0.5 | 49-25 | 79-65 |
| Level four | <1.0 | <0.5 | <25 | <65 |

Table S2 Clinical impact of mNGS results on anti-infective treatment.

| **Treatment changes owing to mNGS results** | **No. (%)** |
| --- | --- |
| **No changs** |  |
| Empirical antibiotic treatment continued | 23 (37.10%) |
| **Treatment changes** |  |
| Add antibiotics on the basis of the original | 24 (38.71%) |
| Adjust antibiotics on the basis of the original | 14 (22.58%) |
| Reduce antibiotics on the basis of the original | 1 (1.61%) |

Table S3 Changes in patient indicators within the subsequent 7 days after optimizing anti-infective treatment.

|  | Improvement | No improvement |
| --- | --- | --- |
| White blood cell (10^9^/L) | 35 (56.45%) | 27 (43.55%) |
| C reactive protein (mg/L) | 41 (66.13%) | 21 (33.87%) |
| Procalcitonin (ng/mL) | 39 (62.90%) | 23 (37.10%) |
| Oxygenation index (mmHg) | 50 (80.65%) | 12 (19.35%) |
| Chest X-ray or CT | 32 (51.61%) | 30 (48.39%) |
